# Efficacy of fluralaner spot-on solution against induced infestations with *Rhipicephalus sanguineus* on dogs

**DOI:** 10.1186/s13071-016-1523-4

**Published:** 2016-05-31

**Authors:** Janina Taenzler, Julian Liebenberg, Machiel Mienie, William R. Everett, David R. Young, Thomas S. Vihtelic, Fangshi Sun, Eva Zschiesche, Rainer K. A. Roepke, Anja R. Heckeroth

**Affiliations:** MSD Animal Health Innovation GmbH, Zur Propstei, 55270 Schwabenheim, Germany; ClinVet International, Uitsigweg, Bainsvlei, 9338 Bloemfontein, Free State South Africa; MSD Animal Health Malelane Research Unit, PO Box 124, Malalane, 1320 Republic of South Africa; BerTek, Inc., 104 Wilson Bottoms Road, Greenbrier, AR 72058 USA; Young Veterinary Research Services, 7243 East Avenue, Turlock, CA 95380 USA; MPI Research, 54943 North Main Street, Mattawan, MI 49071 USA; Merck Animal Health, 2 Giralda Farms, Madison, NJ 07940-1026 USA

**Keywords:** Bravecto™ Spot-on Solution, Dog, Efficacy, Fluralaner, *Rhipicephalus sanguineus*, Tick

## Abstract

**Background:**

The efficacy of fluralaner spot-on solution administered once topically against induced infestations with *Rhipicephalus sanguineus* was evaluated in dogs over a 12-week post-treatment period.

**Methods:**

Six negative-controlled studies were conducted, involving a total of 112 adult dogs (57 mixed breed, 47 Beagles, eight Labradors). In each study, dogs were randomized to two groups of eight to ten dogs each. On day 0, dogs in each treated group were topically administered fluralaner spot-on solution once at a dose of 25 mg/kg body weight, while dogs in each control group were not treated. Two days before treatment, and on days 28, 56 and 84 after treatment, all dogs were infested with approximately 50 unfed, adult *Rh. sanguineus* ticks (sex ratio 1:1). Ticks were removed and counted on days 2, 30 (4 weeks), 58 (8 weeks), and 86 (12 weeks) after treatment to assess efficacy.

**Results:**

Efficacy against ticks 2 days after treatment was 91.1 % (study 1), 98.4 % (study 2), 100 % (study 3), 97.6 % (study 4), 99.6 % (study 5), and 99.8 % (study 6). At all other assessment time points, tick efficacy was 95.4–100 %. Tick reduction in all treatment groups was significant at all assessment time points (*P* < 0.0001).

**Conclusions:**

A single topical administration of fluralaner spot-on solution provides a high level of therapeutic and persistent efficacy against *Rh. sanguineus* ticks over the subsequent 12 weeks.

## Background

*Rhipicephalus sanguineus*, the brown dog tick, is the most widespread tick worldwide [1]. The domestic dog is the main host of this tick in both urban and rural areas [2, 3], but it can also occasionally infest a wide range of domestic and wild hosts, including cats, rodents, birds, and humans [4, 5]. *Rhipicephalus sanguineus* ticks can attach everywhere on the dog, but the head (particularly the ears), interdigital spaces, back, inguinal region and axilla are among their preferred attachment sites [6]. In tropical and subtropical areas, this tick is prevalent throughout the year [6, 7], whereas in temperate regions it is most active from the late spring to early autumn [7]. *Rhipicephalus sanguineus* ticks can even overwinter in the environment and infest dogs during winter in some regions of temperate climate (e.g. southeastern Oklahoma and northwestern Arkansas, United States) [6]. As known vectors of pathogen transmission as well as a cause of discomfort and blood loss for dogs, ticks and tick infestations are a serious concern for owners. Therefore, control of *Rh. sanguineus* is of great medical and veterinary importance.

Over the last decade, the number of products and strategies available for tick control has increased remarkably, offering veterinarians and pet owners the option of choosing their preferred product [8]. Most of the existing commercial tick control products usually have a 4 week tick-killing and/or repellent effect via contact exposure of ticks to the treated animal [9]. An alternative of tick control in dogs is being offered by using drugs from the isoxazoline chemical class (i.e. fluralaner [10], afoxolaner [11] and sarolaner [12]). This alternative exerts its effect when ticks attach to the host and are exposed to the drug through feeding activity [10]. B**y** using fluralaner spot-on solution, a second convenient route of fluralaner administration besides the oral route (Bravecto™) is being presented to veterinarians and pet owners. After topical administration, fluralaner is readily absorbed from the administration site into the skin, from where it is distributed systemically throughout the body. An uptake of the medicated tissue fluid by the *Rh. sanguineus* tick results in a potent tick-killing effect. Pharmacokinetic data suggest that by topical administration of fluralaner 12 weeks of efficacy against *Rh. sanguineus* can be provided [13].

This paper summarizes the results of six independent studies, which evaluated the efficacy of fluralaner spot-on solution against *Rh. sanguineus* ticks over a 12-week post-treatment period.

## Methods

### Study set-up

Six studies (studies 1–6) were conducted in dogs using induced tick infestations with *Rh. sanguineus* after single topical fluralaner (Bravecto™ Spot-on Solution) administration. All studies were in accordance with Good Clinical Practice (VICH guideline GL9, Good Clinical Practice, EMA, 2000). Three studies were conducted in South Africa (studies 1–3) and three studies were conducted in the United States (studies 4–6). All studies were randomized and performed as either negative-controlled and partly masked (studies 1, 4–6), or open-labelled (studies 2 and 3) (Table 1). Each study was approved by the corresponding local Ethics Committee responsible for the respective CRO before study start.

**Table 1:** Study details

Study set-up	Study site	Animals per group		Dog breed	Weight (kg)	Age (years)	Tick isolate used
Study number		Treatment group	Control group				
1	ClinVet (South Africa)	10	10	Mixed and Beagles	8.0–20.2	0.9–6	EU
2	Malelane Research Unit, MSD Animal Health (South Africa)	8	8	Mongrels and Labradors	10.7–35.6	3–6	SA
3	Malelane Research Unit, MSD Animal Health (South Africa)	8	8	Mongrels and Labradors	11.3–29.9	2–6	SA
4	BerTek, USA	10	10	Mixed	6.8–14.9	2.4–11.3	USA
5	Young Veterinary Research Services, USA	10	10	Beagles	7.7–16.3	5.5–6.6	USA
6	MPI Research, USA	10	10	Beagles	4.3–8.7	≥0.5	USA

*Abbreviations*: *EU* Europe, *SA* South Africa, *USA* United States

In total, 112 adult dogs (57 mixed breed, 47 Beagles, eight Labradors), consisting of males and females between 0.5 and 11 years of age, and weighing between 4.3 and 35.6 kg before treatment, were involved in the studies. Each dog was in good health, had not been treated with any parasite control product within 2 to 3 months prior to a 7-day acclimatization period, did not harbor any ticks before treatment, and was uniquely identified by a tattoo or a microchip number.

Prior to randomization, dogs were clinically examined and weighed. The parasite susceptibility of each dog was confirmed through the infestation with approximately 50 unfed, adult *Rh. sanguineus* ticks (sex ratio 1:1), followed by tick removal and count 48 h (± 4 h) later.

All dogs, from every study group, harbored more than 25 % of the number of originally infested ticks. Dogs were blocked by descending live tick counts and one dog in each block was randomized to two study groups (one treatment and one control group) of eight dogs (studies 2 and 3) or ten dogs (studies 1, 4–6), using a computer generated randomization list.

All dogs were kept indoors and housed individually during the study course. They were fed a commercially obtained dry dog food at least once daily at the recommended rate; drinking water was provided *ad libitum*. General health observations were performed once daily throughout the study.

### Treatment

On day 0 (i.e. day of treatment), dogs in the treatment group were treated with fluralaner spot-on solution (Bravecto™ Spot-on Solution), based on the dog’s individual body weight, to achieve a dose of 25 mg fluralaner/kg body weight. Between 0.4 and 3.2 ml of fluralaner spot-on solution/dog was administered once topically at one or more spots, depending on the administration volume, along the dog’s dorsal line. During administration, the dog was standing, or lying with its back horizontally. The first spot was administered between the shoulder blades and the other spots were administered approximately 5–10 cm caudal of the previous spot. The hair was parted, the tip of a positive displacement pipette (studies 2 and 3) or of a disposable syringe (studies 1, 4–6) was placed vertically on the skin, and the solution was administered directly to the skin by pressing the plunger of the syringe to empty its contents. After administration, the dog was held upright for approximately 5 min to observe the treatment site. No run or drip-off was observed and no additional treatment solution was applied to any dog. Dogs in the control group remained untreated.

### Tick infestations and assessments

Infestations using laboratory reared *Rh. sanguineus* ticks were conducted on sedated dogs (study 1) or non-sedated dogs (studies 2–6) 2 days before treatment (i.e. day -2), and on days 28 (4 weeks), 56 (8 weeks), and 84 (12 weeks) after treatment. Dogs in studies 4–6 were not sedated prior to each infestation, but were placed in an infestation chamber for approximately 4 h (± 10 min) to ensure that the ticks had established an infestation. After the infestation period, the dog was released back to its kennel. Dogs in studies 1–3 were infested in their kennel.

The tick isolate used in each study for each infestation was not older than 10 years and ticks were regularly added from the field (Table 1). At each infestation time point, each dog was infested with 50 (± 4) viable, unfed adult ticks (sex ratio 1:1), applied directly to its fur along the dorsal rump area, which allowed the ticks to crawl into the hair coat and select an attachment site.

Tick removal and counts were performed 2 days after treatment (i.e. on day 2) and then 48 h (± 4 h) after each infestation (i.e. on days 2, 30, 58 and 86), and were classified as dead or alive, attached or unattached, and engorged or unengorged. In studies 1, 4, 5 and 6 personnel conducting tick classification and tick counting were masked to the treatment status of each dog.

### Efficacy evaluation

The statistical analysis was performed using the software package SAS® (SAS Institute Inc., Cary, NC, USA, release 9.1.3 (study 2), 9.2 (studies 1 and 3) and 9.3 (studies 4–6). The individual dog was the experimental unit in all statistical calculations. The percentage efficacy against ticks was calculated for the treatment group at each assessment time point using geometric means with Abbott’s formula:

Efficacy (%) = 100 × (M_C_ - M_T_)/M_C,_ where M_C_ is the mean number of total live ticks on untreated control dogs and M_T_ is the mean number of total live ticks on treated dogs. Log-transformed [ln(x + 1)] counts of live attached ticks were used to confirm the efficacy calculation. Significant differences were assessed between the log-counts of live ticks in the treated group at each assessment time point and the log-counts of the untreated control group. Study groups were compared using a linear mixed model, which included study group as a fixed effect and block as a random effect. The two-sided level of significance was set *P* ≤ 0.05.

## Results

Two days after a single topical treatment with fluralaner spot-on solution, efficacy rates of 91.1 % (study 1), 98.4 % (study 2), 100 % (study 3), 97.6 % (study 4), 99.6 % (study 5), and 99.8 % (study 6) were achieved against *Rh. sanguineus* ticks (i.e. therapeutic efficacy). The persistent efficacy against *Rh. sanguineus* ticks, at the assessment time points 30, 56 and 86 in the different studies varied between 95.4 and 100 %. Tick reduction in all treatment groups was significant at all assessment time points (*P* < 0.001) (Table 2).

**Table 2:** Mean tick counts and efficacy (%) after a single topical fluralaner administration (Bravecto™ spot-on solution) against *Rh. sanguineus* infestations in dogs

Assessment time points ^a^		Study day 2		Study day 30		Study day 58		Study day 86	
Study		Fluralaner	Control	Fluralaner	Control	Fluralaner	Control	Fluralaner	Control
1	Mean^b^ tick counts (*n*)	1.8 (3.8)	20.5 (25.1)	0 (0)	29.1 (29.8)	0 (0)	32.6 (32.9)	0 (0)	33.8 (34.0)
	Count range (*n*)	0–19	4^e^–44	0	20–39	0	26–39	0	29–39
	Efficacy^c^ (%)	91.1 (84.9) ^d^		100 (100) ^d^		100 (100) ^d^		100 (100) ^d^	
	*P*-value	0.0004		< 0.0001		< 0.0001		< 0.0001	
2	Mean^b^ tick counts (*n*)	0 (0)	18.7 (19.9)	0 (0)	13.8 (16.5)	0 (0)	24.7 (26.3)	0.1 (0.1)	34.2 (35.8)
	Count range (*n*)	0	10^f^–34	0	3^f^–34	0	16–37	0–1	26–67
	Efficacy ^c^ (%)	100 (100) ^d^		100 (100) ^d^		100 (100) ^d^		100 (100) ^d^	
	*P*-value	< 0.0001		< 0.0001		< 0.0001		< 0.0001	
3	Mean^b^ tick counts (*n*)	0 (0)	10.4 (14.3)	0 (0)	24.3 (25.5)	0 (0)	15.7 (17.9)	0 (0)	19.8 (23.3)
	Count range (*n*)	0	4^g^–37	0	14–38	0	6^g^–26	0	6^g^–35
	Efficacy ^c^ (%)	100 (100) ^d^		100 (100) ^d^		100 (100) ^d^		100 (100) ^d^	
	*P*-value	< 0.0001		< 0.0001		< 0.0001		< 0.0001	
4	Mean^b^ tick counts (*n*)	0.9 (2.8)	37.4 (38.6)	0 (0)	26.1 (27.7)	0.2 (0.3)	18.1 (19.6)	0.6 (1.1)	19.4 (20.8)
	Count range (*n*)	0–23	24–51	0	16–41	0–2	8^h^–33	0–5	12^h^–32
	Efficacy ^c^ (%)	97.6 (92.7) ^d^		100 (100) ^d^		98.9 (98.5) ^d^		96.7 (94.7) ^d^	
	*P*-value	< 0.001		< 0.001		< 0.001		< 0.001	
5	Mean^b^ tick counts (*n*)	0.7 (1.8)	21.1 (25.0)	0 (0)	26.4 (26.9)	0 (0)	28.8 (29.8)	0.1 (0.1)	22.3 (23.1)
	Count range (*n*)	0–11	2^i^–40	0	19–37	0	18–43	1–0	13–33
	Efficacy ^c^ (%)	96.6 (92.8) ^d^		100 (100) ^d^		100 (100) ^d^		99.7 (99.6) ^d^	
	*P*-value	≤ 0.001		≤ 0.001		≤ 0.001		≤ 0.001	
6	Mean^b^ tick counts (*n*)	0.1 (0.1)	30.4 (30.8)	0.1 (0.1)	24.0 (24.5)	0.1 (0.1)	17.6 (18.7)	0.0 (0.0)	17.8 (18.3)
	Count range (*n*)	0–1	21–38	0–1	18–31	0–1	12^j^–33	0	11^j^–25
	Efficacy ^c^ (%)	99.8 (99.7) ^d^		99.7 (99.6) ^d^		99.6 (99.5) ^d^		100 (100) ^d^	
	*P*-value	< 0.001		< 0.001		< 0.001		< 0.001	

^a^Assessment of ticks 48 h (± 4 h) after treatment or re-infestation following treatment; ^b^Geometric mean (arithmetic mean); ^c^Calculated based on geometric mean (arithmetic mean); ^d^Log-counts of live ticks from the treatment group were significantly different (*P* ≤ 0.05) from log-counts of the respective untreated control group

^e^ eight from ten dogs adequately infested; ^f^ seven from eight dogs adequately infested (Study day (SD) 2), five from eight dogs adequately infested (SD 30); ^g^ four from eight dogs adequately infested (SD 2), six from eight dogs adequately infested (SD 58), six from eight dogs adequately infested (SD 86); ^h^ eight from ten dogs adequately infested (Study day (SD) 58 and SD 86): ^i^ nine from ten dogs adequately infested (Study day (SD) 2); ^j^ eight from ten dogs adequately infested (SD58), nine from ten dogs adequately infested (SD86)

Adequate tick infestations (at least six animals with a tick attachment rate > 25 %) were achieved in the control groups of studies 1, 4, 5 and 6 at all tick assessment time points. In study 2, at the 30-day assessment time point (4 weeks after treatment), five out of eight control dogs were adequately infested (mean attachment rate 33.0 %) and in study 3, at the 2 day assessment time point, four out of eight control dogs were adequately infested (mean attachment rate 28.5 %). However, as these inadequate infestations only occurred on one occasion in each of the two studies and as the mean attachment rate was > 25 %, the control dog tick infestations were considered to be sufficiently vigorous to provide a valid result.

All dogs were carefully monitored for adverse events throughout the 12-week post-treatment observation period. One dog in study 2 developed slight erythema at the administration site for 24 h after treatment, and another dog in study 2 had mild wheal development at the administration site 2 h after treatment. Both dogs recovered fully without concomitant treatment. No other adverse reactions were observed in these dogs or any other dog in the studies either at the administration site or systemically throughout the study duration.

## Discussion

Fluralaner spot-on solution is the first topically administered ectoparasiticide to provide an extended period of persistent efficacy against ticks and fleas for dogs [14]. Results of the six studies in this paper demonstrate that fluralaner provides excellent therapeutic and persistent efficacy against adult *Rh. sanguineus* ticks over the entire 12-week recommended re-treatment interval following single topical administration. These results are consistent with the pharmacokinetic data reported by Kilp et al. [13].

*Rh. sanguineus* ticks are prevalent in tropical and subtropical regions throughout the entire year and in temperate regions for much of the year [6, 7]. In addition, these ticks can infest homes and kennels with multiple life stages of ticks present in the housings. Therefore, persistent effective control of this tick is of critical importance. Topically administered fluralaner provides 12 weeks of persistent efficacy against *Rh. sanguineus* ticks which means that four treatments will deliver tick protection for approximately 1 year. In comparison, other drugs of the chemical isoxazoline class only provide a maximum of 4-week tick control [11, 12]. The most common cause of treatment failure is reported to be incorrect treatment administration [15], therefore minimizing the number of required administrations can be a factor in reducing the risk of treatment failure.

Topically administered tick repellent treatments may not provide the same level of efficacy in the extremities as can be delivered by systemic treatments [16]. Therefore, topically administration of a systemically active ingredient, in this case fluralaner, provides a high level of efficacy over the entire body surface. As previously noted, *Rh. sanguineus* ticks can show attachment predilection for body sites that may not be as effectively protected following topical administration of a non-systemic active product. For example, ticks may attach between the foot pads of dogs on the ventral surface of the foot, an area that is frequently wet and difficult to protect with an external (non-systemic treatment [17]. Bravecto™ Spot-on Solution combines convenient administration with persistent efficacy over 12 weeks post-treatment.

Treatment safety is a concern for veterinarians in clinical practice. In the six studies there were mild treatment site dermal reactions observed in two treated dogs that resolved within two or 24 h without concomitant treatment. There were no systemic adverse reactions in any of the treated dogs. Therefore, it is expected that topically administered fluralaner will deliver the same strong level of systemic safety observed with orally administered fluralaner [18].

## Conclusions

Fluralaner spot-on solution administered once topically to dogs delivers a very high level of therapeutic and persistent efficacy against *Rh. sanguineus* ticks over the subsequent 12-weeks.
